# Mitral Annular Disjunction Presenting With Ventricular Arrhythmia: A Case Report

**DOI:** 10.7759/cureus.99179

**Published:** 2025-12-14

**Authors:** Oumaima Taoussi, Hajar Rabii, Soukaina Scadi, Fatimazahra Merzouk, Ghali Benouna

**Affiliations:** 1 Cardiology, Mohammed VI International University Hospital, Mohammed VI University of Health Sciences (UM6SS), Casablanca, MAR; 2 Cardiology, Cheikh Khalifa International University Hospital, Mohammed VI University of Health Sciences (UM6SS), Casablanca, MAR

**Keywords:** cardiac magnetic resonance, mitral annular disjunction, non-sustained ventricular tachycardia, papillary muscle fibrosis, ventricular arrhythmia

## Abstract

Mitral annular disjunction (MAD) is an underrecognized structural abnormality increasingly linked to ventricular arrhythmias and sudden cardiac death. We report the case of a patient admitted for palpitations and chest discomfort, in whom a 12-lead surface electrocardiogram captured a short episode of ventricular tachycardia that terminated spontaneously, consistent with non-sustained ventricular tachycardia (VT). Coronary angiography demonstrated normal coronary arteries, prompting further evaluation with cardiac magnetic resonance imaging. CMR revealed a clear systolic separation between the posterior mitral annulus and the left ventricular myocardium, confirming the presence of MAD. It also identified focal papillary muscle fibrosis adjacent to the disjunction, an arrhythmogenic substrate strongly associated with malignant ventricular arrhythmias, with no evidence of ischemia or myocarditis. Left ventricular systolic function was preserved. The patient was started on medical therapy and referred for electrophysiological assessment due to the arrhythmic burden and high-risk structural findings. This case highlights the importance of considering MAD in patients presenting with ventricular arrhythmias despite normal coronary angiography. Multimodality imaging, particularly CMR, plays a pivotal role in identifying both structural and tissue abnormalities, refining risk stratification, and guiding management. Early recognition of MAD enables targeted follow-up, optimized therapy, and timely intervention to reduce the risk of life-threatening outcomes.

## Introduction

Mitral annular disjunction (MAD) is an increasingly recognized structural abnormality characterized by a systolic separation between the posterior mitral annulus and the basal left ventricular myocardium, best identified by echocardiography or cardiac magnetic resonance (CMR) [1]. Once considered a benign anatomical variant, MAD is now understood to play a central role in the arrhythmic mitral valve prolapse syndrome, a contemporary entity encompassing mitral valve prolapse, MAD, ventricular arrhythmias, and an elevated risk of sudden cardiac death [1,2].

Recent studies have shown that MAD is more common than previously appreciated, particularly when systematically evaluated with advanced imaging. Its detection also varies depending on the modality used and on the diagnostic threshold applied. Clinically, patients with MAD may be completely asymptomatic or may present with palpitations, atypical chest discomfort, presyncope, or documented ventricular arrhythmias. This wide spectrum contributes to the current challenges in risk stratification, as only a subset of individuals with MAD exhibit features associated with heightened arrhythmic risk.

The arrhythmogenic substrate associated with MAD is thought to arise from altered mechanical forces at the annular-ventricular junction, leading to myocardial stretch, microtrauma, and progressive scarring. These mechanisms preferentially affect the inferolateral wall and papillary muscles, where localized fibrosis can trigger complex ventricular arrhythmias [2,3]. Late gadolinium enhancement (LGE) on CMR, particularly involving the papillary muscles or adjacent myocardium, has emerged as a key marker of arrhythmic risk in this population [3]. As a result, CMR has become an indispensable tool, not only for confirming the presence and extent of MAD, but also for identifying myocardial fibrosis and refining risk stratification beyond what is possible with echocardiography alone [1,3].

We present a case of non-sustained ventricular tachycardia in a patient with angiographically normal coronary arteries, in whom CMR revealed mitral annular disjunction with focal papillary muscle fibrosis. This case underscores how multimodality imaging can diagnose an underlying arrhythmogenic substrate, guide clinical management, and ultimately help mitigate the risk of life-threatening ventricular arrhythmias.

## Case presentation

**Figure 1:**
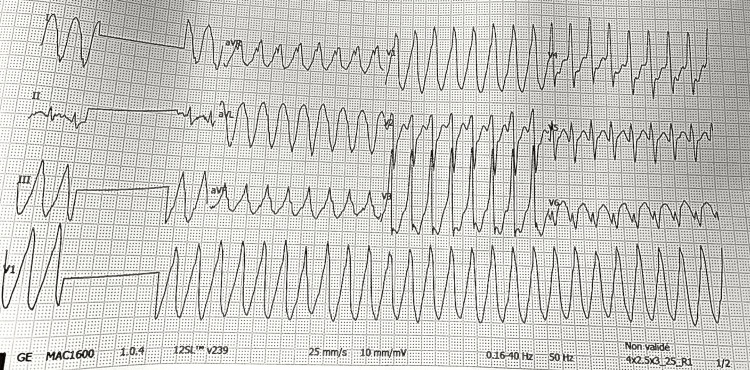
Surface ECG showing non-sustained ventricular tachycardia. An electrocardiogram captured a brief episode of wide-complex tachycardia that terminated spontaneously, consistent with non-sustained ventricular tachycardia (NSVT). Precordial leads (V1–V6) and augmented limb leads (aVR, aVL, aVF) are clearly recorded. Minor limb-lead artifacts are present but do not affect interpretability of the tachycardia morphology.

A 62-year-old male, an active smoker with no other significant past medical history, presented with a one-week history of recurrent palpitations accompanied by intermittent, non-exertional chest discomfort. Upon arrival, he was hemodynamically stable. The admission ECG showed sinus rhythm with frequent premature ventricular contractions (PVCs). During continuous monitoring, a brief self-terminating wide-complex tachycardia episode was recorded, consistent with non-sustained ventricular tachycardia (NSVT) (Figure 1). Precordial leads (V1-V6) and augmented limb leads (aVR, aVL, aVF) were clearly captured, although minor limb-lead artifacts were present without affecting interpretability. A baseline 12-lead ECG was not available; however, no conduction abnormalities, repolarization changes, or QT prolongation were observed during telemetry. Ambulatory rhythm monitoring later demonstrated additional PVCs and short NSVT runs, allowing quantification of the arrhythmic burden.

Given the documented ventricular arrhythmia, a coronary angiography was performed and revealed no obstructive coronary artery disease. To further explore the arrhythmic substrate, a comprehensive cardiac magnetic resonance (CMR) study was obtained. Prior to CMR acquisition, alternative causes of ventricular tachycardia were excluded through laboratory testing, including normal electrolytes, renal function, thyroid profile, and cardiac biomarkers. The absence of ischemic symptoms, together with normal coronary angiography and unchanged telemetry parameters, further reduced the likelihood of ischemia or acute myocardial injury as a trigger. An invasive electrophysiology study was not pursued at this stage, as the patient remained hemodynamically stable and noninvasive imaging was expected to provide sufficient diagnostic clarity.

CMR provided key diagnostic insights. Cine sequences demonstrated preserved biventricular size and systolic function, with a left ventricular ejection fraction of 65% and no regional wall-motion abnormalities. A bi-leaflet mitral valve prolapse (MVP) was present, resulting in moderate eccentric mitral regurgitation (regurgitant volume 18 mL, regurgitant fraction 21%). Most importantly, a systolic separation between the posterior mitral annulus and the basal left ventricular myocardium was identified, confirming mitral annular disjunction (MAD). The characteristic systolic curling motion of the annulus was clearly appreciated on long-axis three-chamber cine images (Video 1).

**Video 1:** Cine three-chamber view demonstrating the dynamic curling motion. Cardiac magnetic resonance cine imaging in the long-axis three-chamber view demonstrating the characteristic systolic posterior displacement of the mitral annulus known as the curling sign which is a dynamic hallmark of mitral annular disjunction.

**Figure 2:**
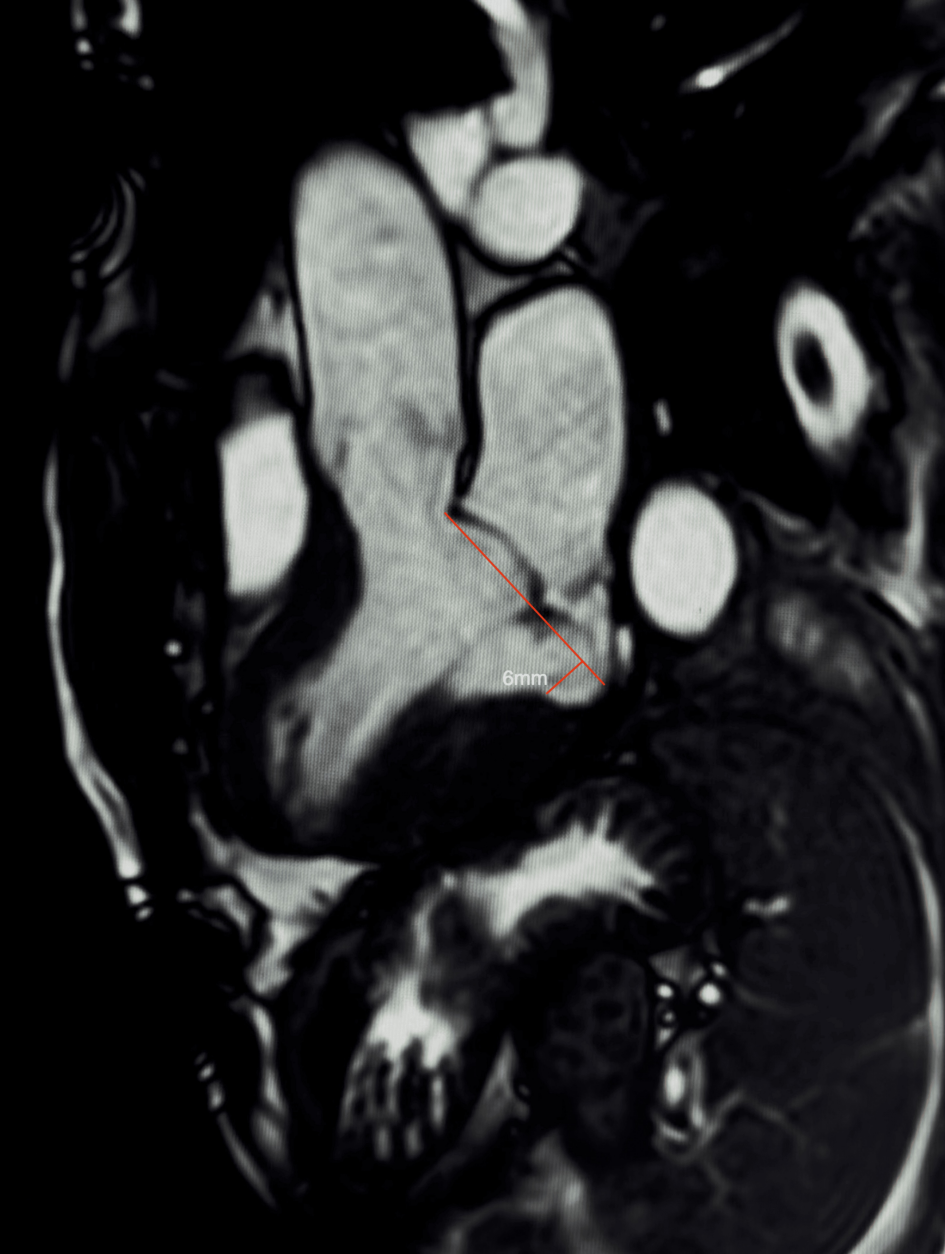
Three-chamber cine frame showing mitral annular disjunction of 6 mm. End-systolic cine still frame in the three-chamber view demonstrating a measurable 6 mm systolic separation between the posterior mitral annulus and the basal left ventricular myocardium, confirming mitral annular disjunction. A subtle bi-leaflet mitral valve prolapse is also visible relative to the annular hinge point.

A static end-systolic frame precisely illustrated the measurable disjunction and subtle prolapse relative to the annular hinge point (Figure 2).

**Figure 3:**
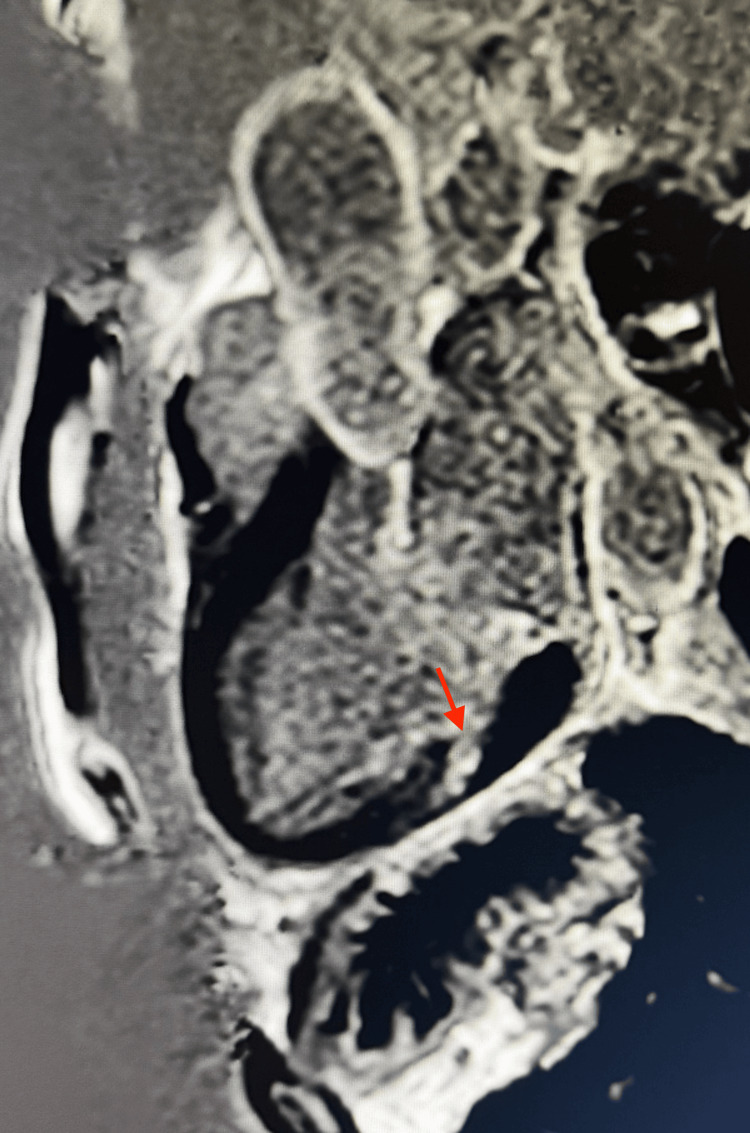
Late gadolinium enhancement imaging revealing papillary muscle fibrosis. Three-chamber late gadolinium enhancement (LGE) image demonstrating focal enhancement of the anterolateral papillary muscle along with a mid-myocardial stripe of enhancement in the adjacent inferolateral wall, findings consistent with a non-ischemic arrhythmogenic substrate.

Tissue characterization refined the arrhythmic risk assessment. Native T1 values were elevated within the anterolateral papillary muscle and the adjacent inferolateral wall, consistent with interstitial or replacement fibrosis. T2 mapping and STIR sequences showed no myocardial edema. Following gadolinium administration, late gadolinium enhancement (LGE) imaging demonstrated focal fibrosis of the anterolateral papillary muscle, along with a mid-myocardial stripe of enhancement in the inferolateral wall, a pattern incompatible with ischemia or myocarditis and strongly suggestive of an arrhythmogenic substrate (Figure 3).

Taken together, mitral annular disjunction, papillary muscle fibrosis, inferolateral mid-wall enhancement, and documented NSVT, the findings supported a diagnosis of arrhythmic mitral valve prolapse syndrome. The patient was started on beta-blocker therapy and referred urgently to a cardiac electrophysiology service for comprehensive sudden cardiac death (SCD) risk stratification. A close follow-up plan was established, including ambulatory rhythm monitoring to quantify the arrhythmic burden and assess therapeutic response. The indication for a primary-prevention implantable cardioverter-defibrillator (ICD) will be further re-evaluated based on the patient’s clinical evolution and updated risk profile.

## Discussion

MAD is increasingly recognized as an arrhythmogenic phenotype, particularly when it coexists with bi-leaflet mitral valve prolapse (MVP). Rather than representing a benign anatomical variant, MAD modifies annular mechanics, leading to excessive systolic excursion and traction on the papillary muscles and basal inferolateral myocardium. Over time, these mechanical stresses may contribute to microstructural injury and focal fibrosis, providing a substrate for ventricular ectopy, NSVT, and, in some reported cases, sudden cardiac death (SCD) [2,3]. Electrocardiographic findings such as PVCs of papillary muscle origin, NSVT, and inferolateral repolarization abnormalities have been associated with arrhythmic phenotypes in MVP/MAD cohorts [4,5]. Importantly, arrhythmic events may occur despite preserved ejection fraction and only mild-to-moderate mitral regurgitation, suggesting that electrical instability does not necessarily correlate with hemodynamic severity [2,6].

Cardiac magnetic resonance (CMR) plays a central role in the evaluation of MAD by enabling both morphological assessment and tissue characterization. Cine imaging allows quantification of the disjunction and visualization of the dynamic curling motion, often associated with arrhythmic forms of the disease [1,3]. Late gadolinium enhancement (LGE), particularly involving the papillary muscles or inferolateral myocardium, has been associated with higher arrhythmic risk in observational studies [2,6,7]. Although the presence of LGE does not establish causality on its own, distinguishing MAD without fibrosis, typically associated with a more benign course, from MAD with focal scarring provides meaningful information for individualized risk assessment. In our patient, papillary muscle fibrosis and inferolateral mid-wall enhancement align with imaging features described in higher-risk subsets.

The growing integration of consumer wearables adds a complementary dimension to arrhythmia detection in this population. Smartwatches equipped with photoplethysmography (PPG) and single-lead ECG capabilities may identify intermittent ventricular ectopy or tachyarrhythmias that conventional short-term monitoring could miss. Recent case reports, including a 2025 publication describing smartwatch-detected ventricular arrhythmias in a patient with MAD [8], suggest potential value as adjunctive tools for rhythm surveillance. Although not a substitute for medical-grade monitoring, these devices may support timely evaluation in selected patients with episodic symptoms.

Management of MAD-associated arrhythmias remains challenging in the absence of formal guidelines. Beta-blockers are frequently used to reduce adrenergic triggers, although their effect on long-term outcomes remains uncertain. Expert consensus emphasizes a personalized approach integrating clinical symptoms, arrhythmia burden, MAD morphology, leaflet involvement, and tissue characterization findings [7]. The role of implantable cardioverter-defibrillators (ICDs) for primary prevention remains controversial. While ICD implantation is clearly indicated for secondary prevention, the 2022 EHRA consensus states that patients with recurrent NSVT, unexplained syncope, or high-risk imaging features, such as papillary muscle or inferolateral fibrosis, may warrant consideration [7]. Several authors have suggested that combinations such as MAD, bi-leaflet prolapse, fibrosis on LGE, and documented NSVT, features all present in our patient, may indicate a phenotype that benefits from early electrophysiologic assessment and careful ICD discussion [2,3,6].

In this patient, the coexistence of structural abnormalities (MAD and bi-leaflet prolapse), tissue abnormalities (papillary muscle and inferolateral fibrosis), and documented NSVT supports classification within a higher-risk arrhythmic MVP/MAD spectrum. A management plan combining beta-blocker therapy, electrophysiology referral, ambulatory rhythm monitoring, smartwatch-assisted rhythm tracking, and periodic reassessment of ICD candidacy is appropriate. Ongoing arrhythmic or structural progression would further strengthen the indication for primary-prevention ICD implantation.

## Conclusions

Mitral annular disjunction represents an important arrhythmogenic substrate, primarily when associated with myocardial fibrosis and ventricular ectopy. In this case, the association of structural abnormalities and documented NSVT identified a higher-risk profile despite preserved ventricular function. Cardiac magnetic resonance was essential for uncovering the underlying fibrotic substrate. In the absence of formal management guidelines, a personalized approach including electrophysiology referral and close longitudinal follow-up is crucial to assess the potential need for primary-prevention ICD therapy.
